# Mutation of *Semaphorin-6A* Disrupts Limbic and Cortical Connectivity and Models Neurodevelopmental Psychopathology

**DOI:** 10.1371/journal.pone.0026488

**Published:** 2011-11-21

**Authors:** Annette E. Rünker, Colm O'Tuathaigh, Mark Dunleavy, Derek W. Morris, Graham E. Little, Aiden P. Corvin, Michael Gill, David C. Henshall, John L. Waddington, Kevin J. Mitchell

**Affiliations:** 1 Smurfit Institute of Genetics and Institute of Neuroscience, Trinity College Dublin, Dublin, Ireland; 2 Molecular and Cellular Therapeutics, Royal College of Surgeons in Ireland, Dublin, Ireland; 3 Physiology and Medical Physics, Royal College of Surgeons in Ireland, Dublin, Ireland; 4 Neuropsychiatric Genetics Research Group, Institute of Molecular Medicine and Department of Psychiatry, Trinity College Dublin, Dublin, Ireland; Tokyo Medical and Dental University, Japan

## Abstract

Psychiatric disorders such as schizophrenia and autism are characterised by cellular disorganisation and dysconnectivity across the brain and can be caused by mutations in genes that control neurodevelopmental processes. To examine how neurodevelopmental defects can affect brain function and behaviour, we have comprehensively investigated the consequences of mutation of one such gene, *Semaphorin-6A*, on cellular organisation, axonal projection patterns, behaviour and physiology in mice. These analyses reveal a spectrum of widespread but subtle anatomical defects in *Sema6A* mutants, notably in limbic and cortical cellular organisation, lamination and connectivity. These mutants display concomitant alterations in the electroencephalogram and hyper-exploratory behaviour, which are characteristic of models of psychosis and reversible by the antipsychotic clozapine. They also show altered social interaction and deficits in object recognition and working memory. Mice with mutations in *Sema6A* or the interacting genes may thus represent a highly informative model for how neurodevelopmental defects can lead to anatomical dysconnectivity, resulting, either directly or through reactive mechanisms, in dysfunction at the level of neuronal networks with associated behavioural phenotypes of relevance to psychiatric disorders. The biological data presented here also make these genes plausible candidates to explain human linkage findings for schizophrenia and autism.

## Introduction

There is compelling evidence that many psychiatric disorders have their origins in disturbed neurodevelopment [1], [2], [3]. Widespread cellular disorganization as well as long-range structural dysconnectivity in schizophrenia (SZ) [4], [5], [6], bipolar disorder (BD) [7], [8], [9] and autism spectrum disorders (ASD) [10], [11], [12], [13], [14] are consistent with primary defects in cell migration, axon guidance and/or synaptogenesis in many brain areas. These findings are also consistent with symptoms in these disorders across many psychological domains and brain systems (cognitive, affective, social, motor and perceptual). There is considerable shared genetic risk across these disorders [15], [16], [17], as well as epilepsy [18], [19] and mental retardation [20]. These disorders can be caused by single mutations in any of a large number of loci [17], [20], [21], [22], [23], and many of the putatively causal mutations predispose to more than one disorder [20]. Strikingly, many of the genes implicated have crucial roles in neurodevelopmental processes [20], [22], [24], [25], [26], [27]. These various disorders may thus represent more or less distinct phenotypic endpoints arising from common neurodevelopmental insults.

Collectively, the genes so far identified explain only a small fraction of disease cases. Evolutionary genetic theory suggests there must be many such genes that can be mutated to cause disorders such as SZ, in order to maintain the high prevalence of the disorder in the face of strong negative selection [23], [28]. Other neurodevelopmental genes are thus good *a priori* candidates to contribute to the etiology of psychiatric disorders.

From this perspective, genes in the semaphorin and plexin families emerge as promising candidates [29]. The transmembrane semaphorin Semaphorin-6A (Sema6A) interacts with the transmembrane proteins Plexin-A2 and Plexin-A4, and, along with Sema6B, these proteins co-ordinately control axon guidance [30], [31], laminar connectivity [32], [33], [34], neuronal migration [35], [36] and dendrite development [37]. Mutation of *Sema6A* results in widespread but subtle derangements of cytoarchitecture and neuronal connectivity in various parts of the brain [30], [31], [32], [33], [36], [38], [39], [40].

Semaphorin genes have been previously implicated in psychiatric disorders. In humans, variants in *PLXNA2* [41], [42], [43] have been associated with risk for SZ and variants in *SEMA6A* with risk for ASD [44]. Furthermore, alterations in expression levels of multiple semaphorins, plexins or semaphorin signalling proteins have been observed in the cortex of post mortem schizophrenia [45], [46] or autism [47] patient brains [45], [46] and in animal models of NMDA-receptor blockade [48], which model psychosis in humans.

In this paper, we provide a comprehensive survey of neuroanatomical defects in *Sema6A* mutant mice, which include previously unreported limbic and cortical cellular disorganisation and dysconnectivity. Some of these changes resemble the reported neuropathology in ASD and SZ. We also characterise these animals ethologically and in a broad panel of behavioural tests and analyse global neural activity patterns using electroencephalography. We find that *Sema6A* mutants display electrophysiological and behavioural phenotypes that phenocopy some of the defects observed in accepted animal models of SZ and that can be reversed by antipsychotics. We consider these results in light of association and linkage findings in humans for loci encoding SEMA6A and interacting proteins.

## Results

### Neuroanatomical phenotypes in Sema6A mutant mice

As neurodevelopmental mutations typically affect multiple brain regions, any of which might contribute to behavioural or physiological phenotypes in mice or to the broad array of symptoms in humans, we set out to comprehensively characterize the anatomical defects across the brain due to *Sema6A* mutation. We were particularly interested, however, in areas most strongly implicated in the psychopathology of psychiatric disorders, including the prefrontal cortex and limbic system, which had not been previously investigated in these mutants.

#### Prefrontal cortex

**Figure 1:**
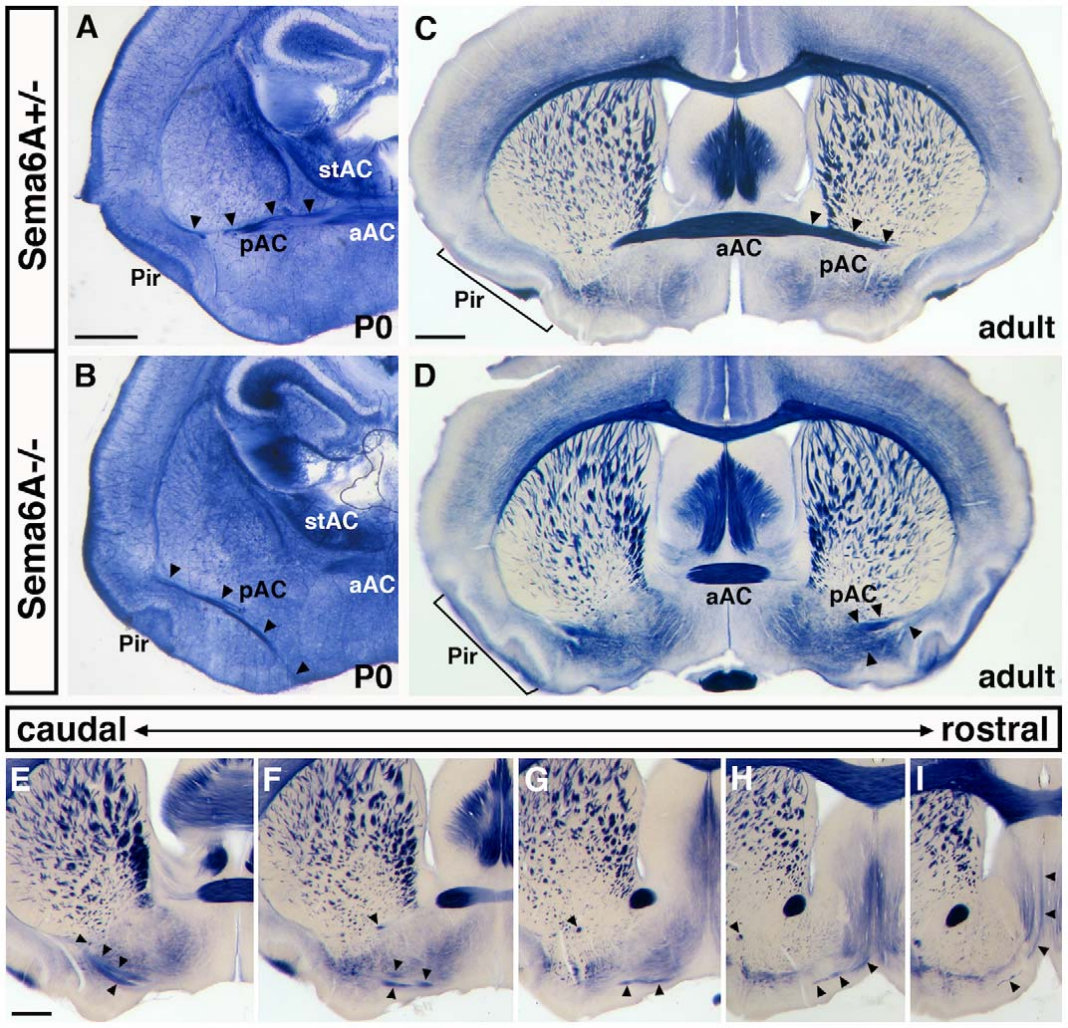
Prefrontal cortex dysconnectivity in *Sema6A* mutants. We visualized pAC projections using the placental alkaline phosphatase (PLAP) marker encoded in the *Sema6A* mutant allele [38]. During development, PLAP is expressed in the neurons that form the pAC (Figure 1A, B), while in adults, the PLAP marker is expressed by oligodendrocytes and thus labels all myelinated axons (Figure 1C, D). In newborn *Sema6A^−/−^* mice (B), the PLAP-stained pAC axons travel ventrally into hypothalamic areas and fail completely to cross the midline as seen in *Sema6A^+/−^* mice (A). Such misrouted pAC axons are still present in adult *Sema6A^−/−^* brains (C, compare with *Sema6A^+/−^* in D) and can be found through extended levels of the caudo-rostral axis within inappropriate regions, such as the septum (E-I, arrowheads). The aAC and stAC are normal in young and adult *Sema6A^−/−^*.a/p/stAC: anterior/posterior/stria terminalis arm of the anterior commissure; Pir: piriform cortex; Scale bar in (A) is for (A, B), in (C) is for (C, D), and in (E) is for (E–I): 500 µm.

The prefrontal cortex in rodents encompasses the agranular insular cortex, the orbitofrontal cortices and some of the cingulate cortices [49]. Some neurons in these areas normally send axons to the opposite hemisphere across the posterior limb of the anterior commissure (pAC). In embryonic (E16–17.5, n = 3, not shown) and newborn (postnatal days (P)0–2, n = 9) Sema6A homozygous mutants, all axons of the pAC project ventromedially rather than medially and fail to cross the midline (Figure 1A,B). Many misrouted pAC axons can be found in adult Sema6A−/− mice (n = 5, Figure 1C,D), where they extend into the hypothalamus and septum (Figure 1E–I). Retrograde tracing of pAC axons in homozygous mutant animals also reveals that the cell bodies of these neurons are ventrally misplaced and located within piriform, rather than insular cortex, where they are observed in control animals (Supporting Information S1).

#### Piriform cortex and olfactory structures

**Figure 2:**
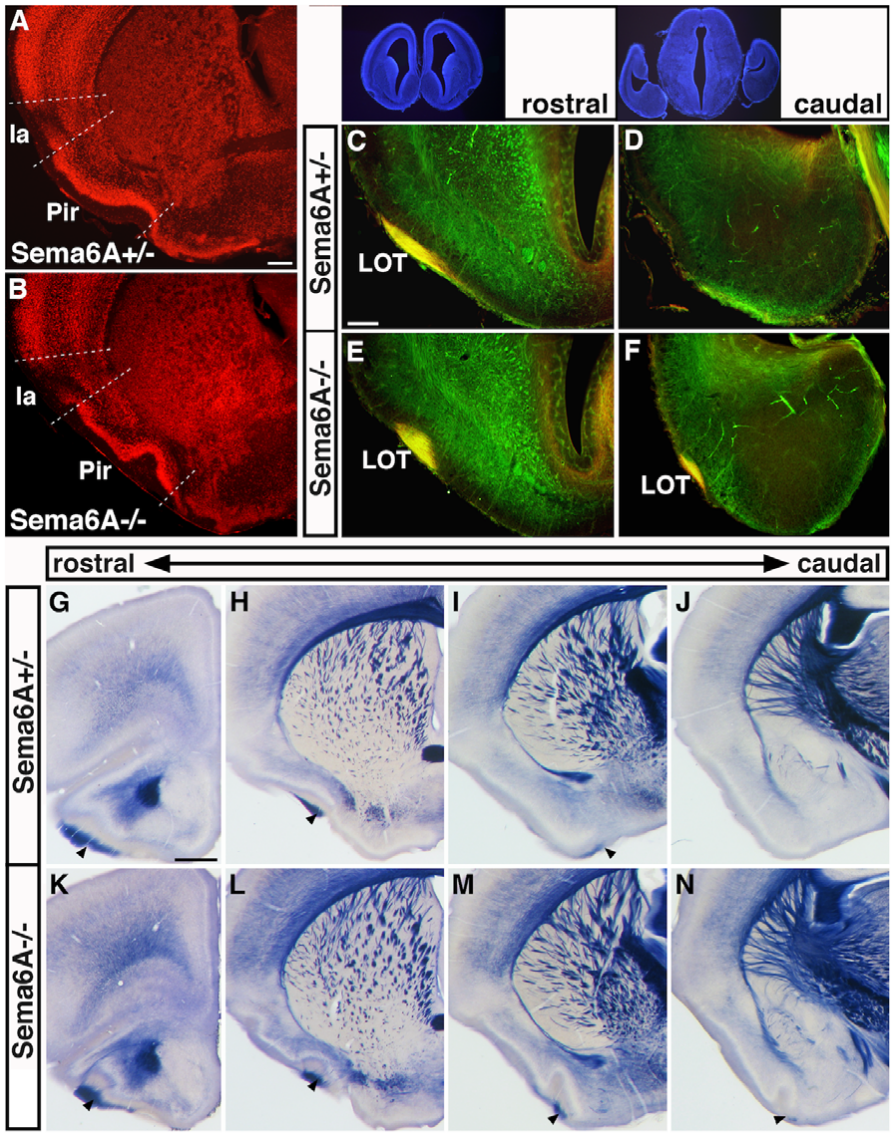
Defects in piriform cortex and olfactory projections. (A, B) NeuN-immunohistochemistry of coronal P10 brains sections demonstrates a greatly exaggerated folding of the piriform cortex in *Sema6A^−/−^* (B), compared to *Sema6A^+/−^* mice (A). (C–F) E16 *Sema6A^+/−^* (C, D) and *Sema6A^−/−^* (E, F) brain sections immunostained for L1 (green) to label all fibres and for Neuropilin-1 (NP-1) to label more specifically LOT axons. In *Sema6A^−/−^* mutants, the L1/NP-1 double-stained LOT (yellow) appears rounded and more embedded into layer 1 of the piriform cortex (E) in contrast to a superficially located LOT in *Sema6A^+/−^* mice (C). In addition, the LOT extends far further caudally than normal (compare F and D). (G–N) Series of PLAP-stained adult brain sections, showing persistence of a more rounded and displaced LOT (arrowheads) that extends further caudally in *Sema6A^−/−^* mutants (K–N) when compared with *Sema6A^+/−^* mice (G–J). Note also that adult *Sema6A^−/−^* mutants show an exaggerated folding of the pirifom cortex, which extends far caudally. Ia: agranular insular cortex; lot: lateral olfactory tract; Pir: piriform cortex. Scale bar in (A) is for (A, B): 200 µm; in (C) is for (C–D): 100 µm; in (G) is for (G–N): 500 µm.

Staining for PLAP, Nissl, or with anti-NeuN antibodies at various ages highlighted a dramatic change in morphology of the piriform cortex in Sema6A homozygous mutants. This three-layered structure receives direct input from the olfactory bulb and connects reciprocally with prefrontal cortex and many other structures [50]. In Sema6A mutants, the folding of the piriform cortex, most readily visualized in the densely packed cells of layer 2, is greatly exaggerated and is evident far further caudally than normal (Figure 2A, B; see also Figure 1A–D and 2G–N). In addition, axons from the lateral olfactory tract (LOT), which normally extend very superficially along the surface of the piriform cortex (Figure 2C, G–I) are displaced internally in Sema6A mutants at E16.5 (Figure 2E, F) and in adulthood (Figure 2K–N) and also extend considerably further caudally (Figure 2D, F, and I–N).

#### Neocortex

**Figure 3:**
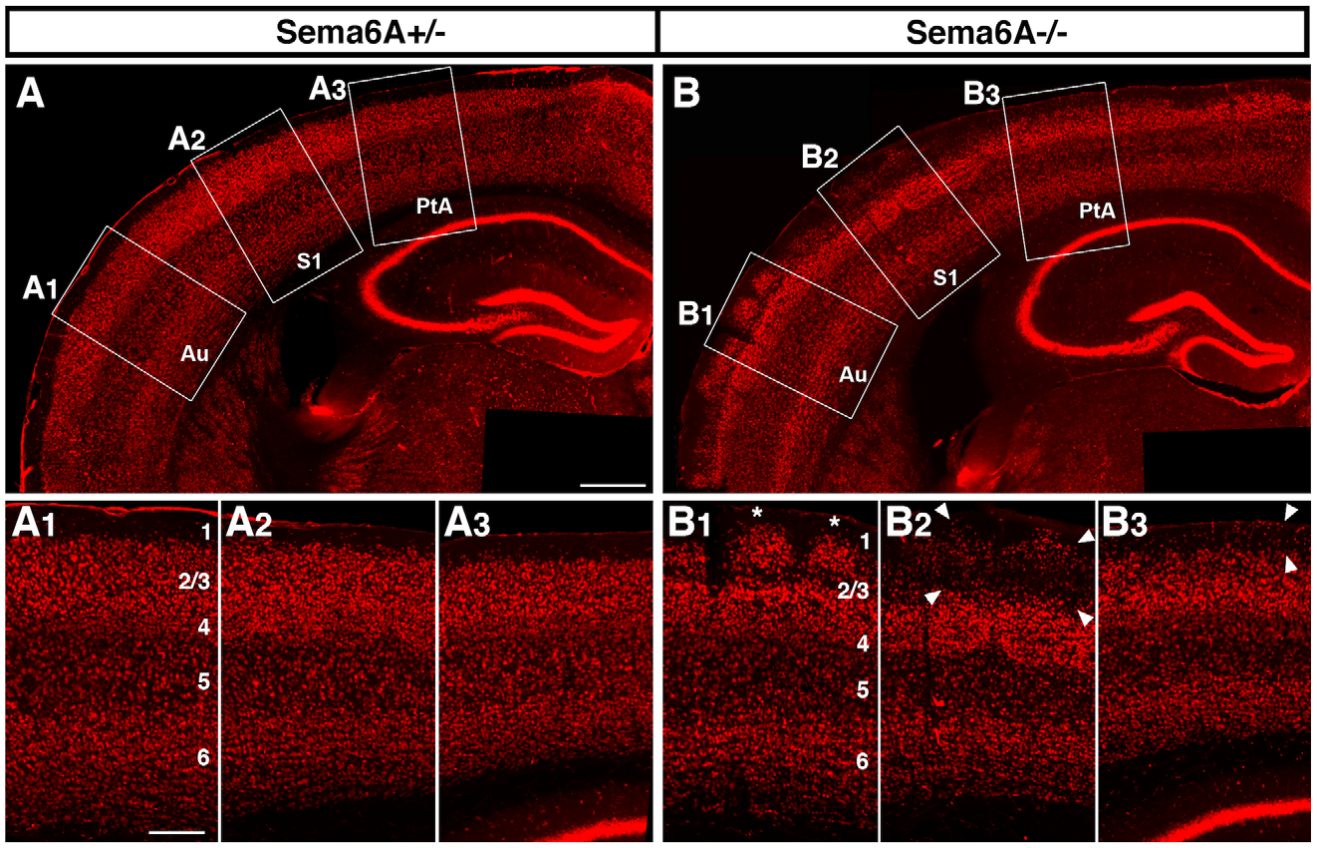
Aberrant lamination of the neocortex. NeuN-immunostaining of coronal sections of adult *Sema6A^+/−^* (A, A1–3) and *Sema6A^−/−^* (B, B1–3) mouse brains in overview (A, B) and detailed view (A1–3, B1–3; position in A, B is indicated as boxes). While the border between layers 1 and 2 of the neocortex is sharply visible in *Sema6A^+/−^* animals (A1–3), it is largely obliterated in *Sema6A^−/−^* mutants (B1–2), and the neuropil of layer 1 appears infiltrated by neurons from deeper layers with a gradient in severity from lateral to medial (B). Laterally, many ectopic neurons form repetitive clusters (asterisks in B1) bordered by neuropil. More medially these neurons are more loosely scattered within the neuropil over a considerable depth (arrowheads in B2, B3). 1–6: Cortical layers 1 to 6. Au: auditory area; S1: primary somatosensory area; PtA: parietal association area. Scale bar in (A) is for (A, B): 500 µm; in (A1) is for (A1–3,B1–3): 200 µm.

Staining with Nissl, anti-NeuN antibodies or DAPI also revealed a defect in the cellular organisation of the neocortex (n = 14 in total; adult: Nissl, n = 4; DAPI, n = 5; NeuN, n = 3; P10: NeuN, n = 2). In Sema6A^−/−^ animals, the normally distinct border between layers 1 and 2 is difficult to distinguish, as many neurons from layers 2/3 invade into the normally cell-sparse neuropil of layer 1 (Figure 3A, B). This defect is not evident at late embryonic stages (E16.5–E17.5) but can be observed from P3 onwards (data not shown).

#### Hippocampal formation

**Figure 4:**
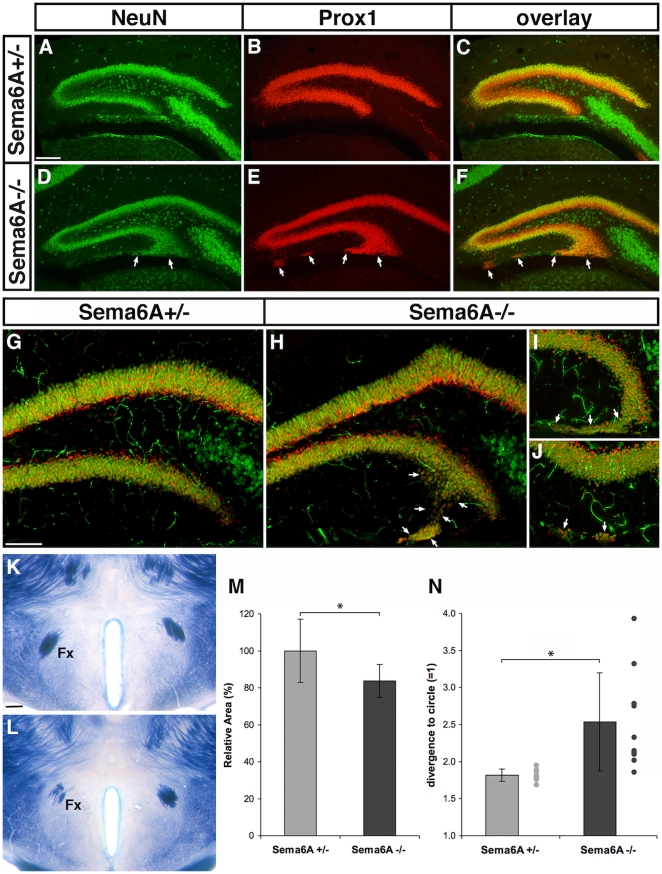
Alterations in hippocampal lamination and projections. (A–F) Immunohistochemistry for NeuN (A, D; green, neurons) and Prox1 (B, E; red, dentate granule cells; overlay in C and F) on coronal brain sections from P10 *Sema6A^+/−^* (A–C) and *Sema6A^−/−^* (D–F) mice. *Sema6A^−/−^* mice show a broadening of the granule cell layer at the tip of the infrapyramidal blade of the dentate gyrus as well as more isolated clusters of ectopic granule cells at the surface of the molecular layer of the same blade (arrows in D–F). (G–H) Overlays of NeuN- (green) and Prox1- (red) immunostained adult brain sections of *Sema6A^+/−^* (G) and *Sema6A^−/−^* (H–J). Similarly distributed ectopic granule cells (arrows in H–J) are still present in the adult dentate gyrus. (K–L) PLAP-stained adult brain sections showing a reduced and defasciculated fornix (Fx) in *Sema6A^−/−^* (L, left fornix) compared with *Sema6A^+/−^* mice (K). The fornix is significantly reduced in size by 20% (M; p<0.05) and is significantly less compact (N; measured as the ratio of actual and circular perimeter; p<0.01) in *Sema6A^−/−^* (n = 10) compared to *Sema6A^+/−^* mice (n = 8). Error bars in (M, N) represent ± SD; *: significant. Dots plotted on the right of columns in (N) are individual data points. Scale bar in (A) is for (A–F), in (G) is for (G–J), and in (K) is for (K, L): 200 µm.

We performed antibody staining for NeuN, a marker of mature neurons, and Prox1, a marker for postmitotic granule cells in the hippocampal dentate gyrus, on brain sections at P10 (Figure 4A–F). In Sema6A^−/−^ mice, we found a subtle but consistent malformation of the infrapyramidal (lower) blade of the dentate gyrus. At the tip of this blade, many misplaced Prox1-positive granule cells were observed, producing a broadened or forked appearance of the tip. In addition, isolated ectopic granule cells were frequently observed within the molecular layer on the infrapyramidal side, some close to the ventricular wall (Figure 4D–F, arrows). These misplaced cells persist in the adult (Figure 4G–J).

**Figure 5:**
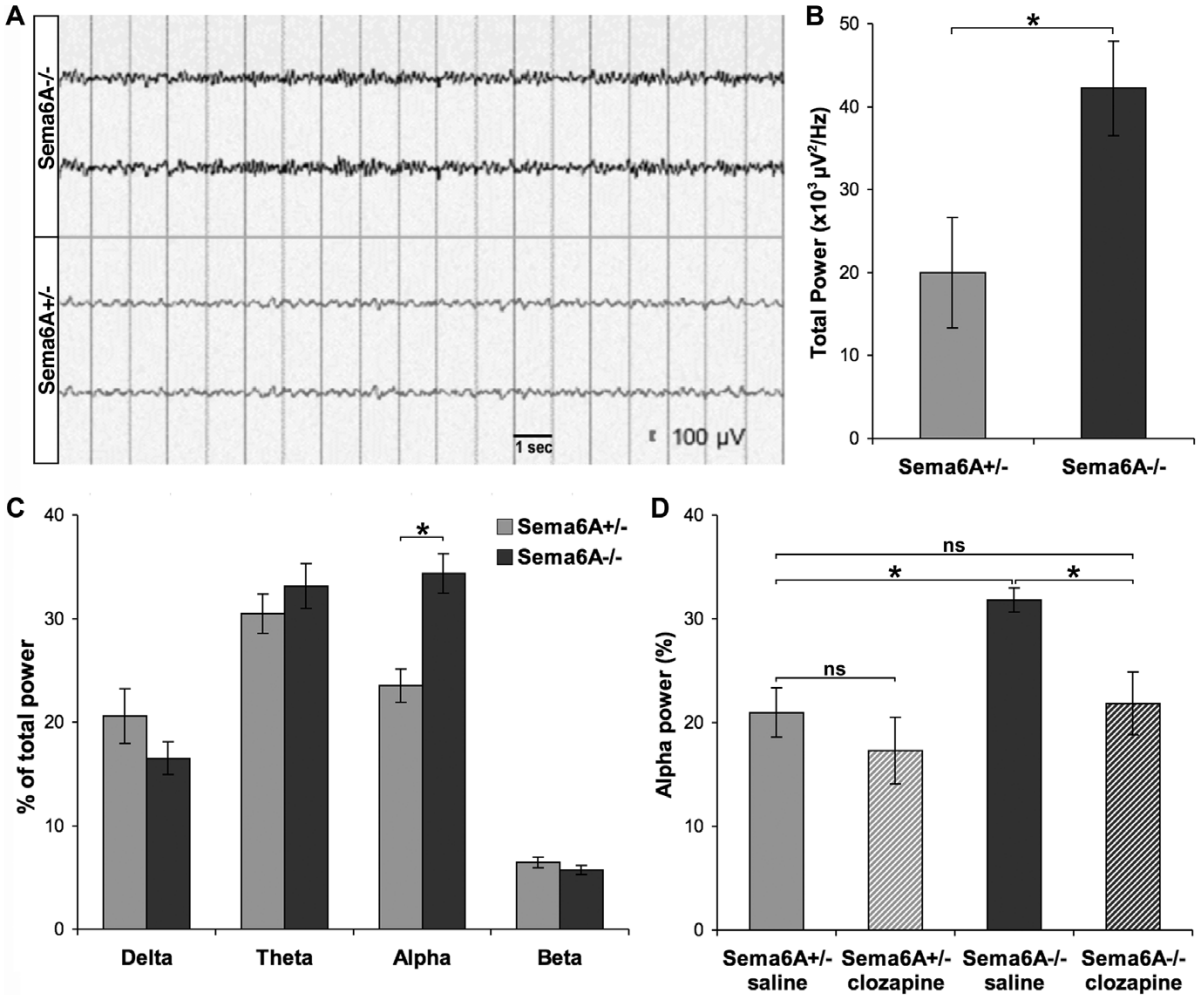
Altered cortical activity patterns. (A) Representative bilateral cortical EEG traces from *Sema6A^−/−^* and *Sema6A^+/−^* animals in spontaneous, freely moving conditions. (B) Quantification of total spectral power in *Sema6A^+/−^* (n = 3) and *Sema6A^−/−^* (n = 4) animals, p<0.005. (C) Quantification of relative power of spectral bands in *Sema6A^+/−^* (n = 7) and *Sema6A^−/−^* (n = 8); a significant increase in the alpha band was observed p<0.001. (D) Increase in relative power in the alpha band in *Sema6A^−/−^* is reversed by administration of clozapine, p<0.05. No significant effect of clozapine on alpha power in *Sema6A^+/−^* animals was observed (n = 4 for all groups). Clozapine at this dose did not affect the other spectral power bands in either genotype (not shown). *p<0.05; ns: non-significant. Error bars represent ± SEM.

We also observed defects in long-range connectivity of the hippocampus. In *Sema6A* mutants, the post-commissural fornix is often defasciculated and reduced in size, sometimes containing far fewer axons than normal. This phenotype is quite variable, even from one side of the brain to the other (Figure 4K, L). On average, the cross-sectional area of the fornix was significantly reduced (p<0.05, T-Test, Figure 4M) to 83.7% (±9 *vs.* 100% ±17.0 in *Sema6A* ^+/−^), as was the degree of compactness (measured by the ratio of actual vs. circular perimeter: 2.53±0.66 in *Sema6A^−/−^ vs.* 1.82±0.08 in *Sema6A^+/−^*; p<0.01, T-Test, Figure 5N).

With the exception of the defect in the fornix and some aspects of the previously reported corticospinal tract defect [30], all the anatomical phenotypes we observe are fully penetrant (i.e., observable qualitatively across all mutant animals analysed).

### Alterations in cortical physiology

We used scalp electroencephalography to examine general levels of cortical activity in *Sema6A* mutants. *Sema6A^−/−^* animals showed a greater spectral density (total power) than *Sema6A^+/−^* mice (42,200±5,668, n = 4 *vs.* 20,010±6,671 µv/Hz, n = 3, respectively; Figure 5A,B) in a cortical electroencephalograph (EEG). Spectral analysis revealed a significant and specific increase in power in the alpha band (8–13 Hz; p<0.001) in *Sema6A^−/−^* compared to *Sema6A^+/−^* animals (Figure 5C). Administration of clozapine (0.25 mg/kg) resulted in normalisation of alpha power to levels comparable to heterozygous controls (Figure 5D), which showed no significant effect of clozapine on alpha power.

### Behavioural analyses

We set out to characterise, in an unbiased fashion, the behavioural consequences of mutation of *Sema6A*, using a broad panel of behavioural tests, along with ethological observation in the home cage environment.

**Figure 6:**
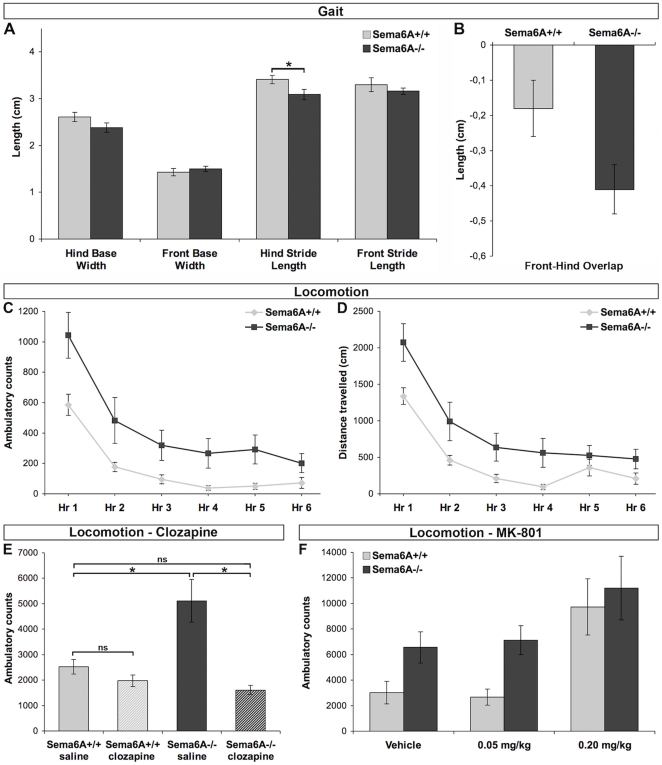
Gait abnormalities and hyperlocomotion. (A) Gait analysis indicated significantly shorter hind-stride length in *Sema6A^−/−^* mutants compared to controls (p<0.05). (B) There is a trend towards decreased hind-front overlap in *Sema6A^−/−^* mutants (p = 0.07). (C) *Sema6A^−/−^* mutants displayed increased number of ambulatory counts in the open-field over a six-hour period (n = 20 per genotype; p<0.05). (D) *Sema6A^−/−^* mutants demonstrated increased exploratory behaviour in the open-field, as indexed by greater distance travelled over a six-hour period (p<0.05). (E) Clozapine (0.25 mg/kg) significantly reversed the hyper-exploratory phenotype in *Sema6A^−/−^* mutants without altering exploration in controls (n = 8 per treatment with vehicle or drug, and per genotype; p<0.05). (F) MK-801-induced hyperlocomotion (0.2 mg/kg) did not differ across the genotypes (n = 8 per treatment with vehicle or drug, and genotype). *p<0.05, **p<0.01; ns: non-significant. Error bars represent ± SEM.

Analysis of gait in *Sema6A^−/−^* mutants revealed a significantly shorter hind stride length in *Sema6A^−/−^* mice relative to wild-type (WT or *Sema6A* ^+/+^) controls (Figure 6A; genotype: F_2, 39_ = 3.24, *P*<0.05, Figure 6A). A marginally significant increase in hind/front overlap was also observed in Sema6a−/− mice (Figure 6B; genotype: F_2, 33_ = 2.89, p = 0.07), as well as a mean decrease in front stride length (WT *vs. Sema6A* ^−/−^: 3.3 cm±0.15 *vs.* 3.16±0.07) and hind base width (WT *vs. Sema6A* ^−/−^: 2.61 cm±0.10 *vs.* 2.38±0.07), although neither of these differences were statistically significant (Figure 6A; p>0.05). Examination of grip strength during the wire hang suspension task failed to reveal any genotypic differences (p>0.05, not shown).

**Figure 7:**
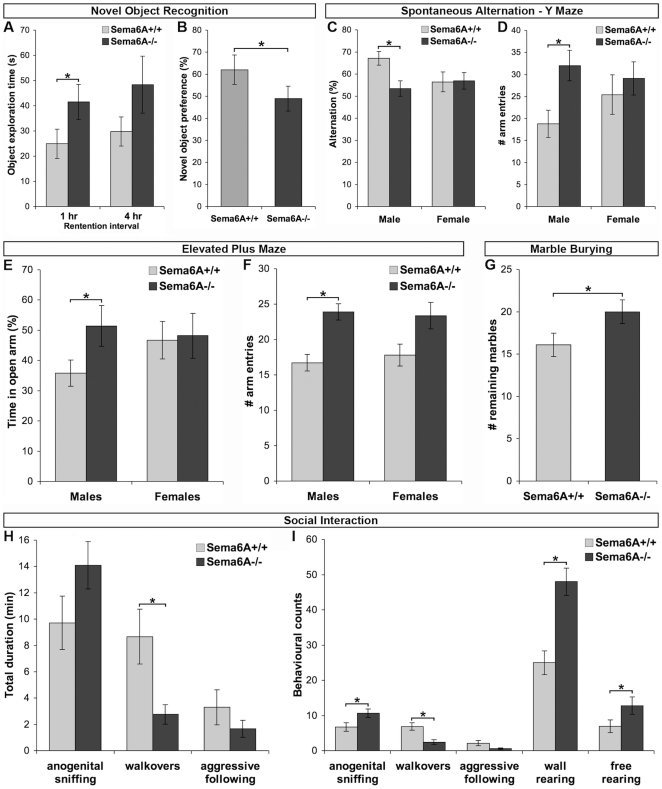
Memory defects, decreased anxiety, and increased social interaction. (A) In the novel object recognition task, *Sema6A^−/−^* mutants demonstrated increased absolute levels of exploration of a novel object at both retention intervals (1 hr, 4 hr; WT: n = 9, *Sema6A* ^−/−^: n = 8; p<0.05). (B) *Sema6A^−/−^* mutants displayed a disruption in novel object recognition memory, as indexed by decreased relative preference to explore the novel object at the 1-hr retention interval, when compared to controls (p<0.05). No interaction with sex was observed for either of these effects. NOR performance at the 4-hr retention interval did not differ from chance (50%) across the genotypes. (C) Spontaneous alternation test of working memory revealed a sex-specific deficit in working memory performance in *Sema6A^−/−^* males (n = 8), as indicated by a decrease in percent alternation, relative to controls (n = 10; p<0.01). (D) A hyper-exploratory phenotype of male *Sema6A^−/−^* mutants was demonstrated by increase in number of overall arm entries in the alternation task relative to controls (p<0.05). (E) In the elevated plus maze test of anxiety, male *Sema6A^−/−^* mutants exhibit decreased anxiety as measured by an increase in the number of open arm entries (WT: n = 10 *Sema6A* ^−/−^: n = 9; p<0.05). (F) *Sema6A^−/−^* mutants demonstrate an increase in number of overall arm entries in the elevated plus maze relative to controls (p<0.01). (G) *Sema6A^−/−^* mutants of both sexes (n = 19) exhibited decreased anxiety in the marble burying task, as indicated by a decreased number of marbles buried compared to controls (n = 22 WT, 19 *Sema6A^−/−^*; p<0.05). (H) Assessment of social interaction with a novel conspecific revealed a decrease in duration of time spent engaged in walkover behaviours (p<0.05) in *Sema6A^−/−^* mutants (n = 11) relative to controls (n = 9). (I) *Sema6A^−/−^* mutants displayed an increase in number of anogenital sniffing (p<0.05) and decrease in walkover (p<0.01) episodes relative to controls. An increase in general exploration of the novel environment was also observed (wall and free rearing). Error bars represent ± SEM.

Examination of open-field exploratory locomotor activity in *Sema6A^−/−^* mice revealed a hyperactive phenotype in both sexes, as indexed by significant increases in activity counts (Figure 6C; genotype: F_2, 46_ = 4.78, p<0.05) and distance travelled (Figure 6D; genotype: F_2, 46_ = 5.65, p<0.05) over a 6-hour observation period. Relative habituation of exploration to the novel environment did not differ between genotypes (genotype×hours interaction: F_10, 230_ = 1.02, p = 0.429). Ethologically-based assessment of exploratory behaviours in a novel environment (the *ethogram* [51]) revealed significant increases in locomotion and rearing in mutants relative to controls (data not shown and Figure 7I). Prior treatment with the antipsychotic clozapine (0.25 mg/kg) reversed the hyper-exploratory phenotype in *Sema6A^−/−^* mice (Figure 6E; genotype×drug interaction: F _4, 67_ = 3.37, p<0.05) to a level indistinguishable from wild-type littermate control mice treated with vehicle or clozapine. Treatment with haloperidol (0.5 mg/kg) reduced control activity count values somewhat, though not significantly (from 2526+/−284 (SEM) in 8 vehicle-treated animals, to 1637+/−545 in 7 haloperidol-treated animals, p = 0.16), but completely reversed the hyperlocomotion in *Sema6A* mutants (from 5116+/−837 to 1556+/−192, n = 8 and 7, respectively, p<0.01). The NMDA-receptor antagonist MK-801 (0.2 mg/kg) stimulated locomotor activity in both *Sema6A^−/−^* mice and controls, with no treatment-genotype interaction; additionally, *Sema6A^−/−^* mice did not show any differential responsiveness to a subthreshold dose of MK-801 (0.05 mg/kg) relative to controls (Figure 6F; genotype×drug treatment interaction; F_4, 56_ = 0.67, p>0.05). Heterozygous animals were not significantly different from wild-type in gait or locomotion tests.

Recognition memory was examined in the Novel Object Recognition (NOR) paradigm. *Sema6A^−/−^* mice demonstrated increased exploration of both objects relative to wild-type controls during the sample phase (WT *vs. Sema6A* ^−/−^: t_14_ = 2.16, p = 0.05) and during test 1 (Figure 7A; WT *vs. Sema6A* ^−/−^: t_14_ = 2.19, p<0.05). *Sema6A^−/−^* mice evidenced impairment in the NOR task, as indexed by decreased exploration of the novel object compared to the familiar object at the 1-hr retention interval (Figure 7B; WT *vs. Sema6A* ^−/−^: t_14_ = 2.18, p<0.05). *Sema6A^−/−^* mice were also examined for spatial working memory in the spontaneous alternation task. Impaired performance, as indicated by decreased alternation across the three arms of the Y maze, was observed in male *Sema6A^−/−^* mice relative to wild-type controls (Figure 7C; t_16_ = 2.98; p<0.01). This was accompanied by an increase in total number of arm entries, a measure of activity levels in this task (WT *vs. Sema6A* ^−/−^: t_16_ = 2.85, p<0.05). These effects were not observed in females.

Emotionality and anxiety-related behaviour were examined in *Sema6A^−/−^* mice in the elevated plus maze. Male *Sema6A^−/−^* mice demonstrated decreased anxiety, as evidenced by increased percent time spent in the open arms relative to the enclosed arms of the maze (Figure 7E; WT *vs. Sema6A* ^−/−^: t_18_ = 2.36, p<0.05). The hyperactive phenotype of *Sema6A^−/−^* mice was also evident in this measure, in terms of an increased number of overall arm entries *vs.* wild-type controls for both sexes (Figure 7E; genotype: F_2, 57_ = 10.304, p<0.0001). No falls from the open arms of the maze were noted for WT or *Sema6A* ^−/−^ animals, indicating no effect of genotype on overall sensorimotor function in this task. When tested in an alternative assay of anxiety, marble-burying behaviour, both male and female *Sema6A^−/−^* mice demonstrated an anxiolytic phenotype, as measured by fewer marbles buried, relative to wild-type controls (Figure 7G; t_39_ = 2.17, p<0.05).

Social interaction, including various affiliative and agonistic behaviours, was assessed in *Sema6A^−/−^* and wild-type controls by placing them with an unfamiliar age-, sex-, and weight-matched C57/BL6 mouse in a novel environment. *Sema6A^−/−^* mice displayed increased social sniffing episodes towards an unfamiliar mouse relative to controls (Figure I; genotype: F_2, 29_ = 7.403, p<0.05). This increase in social investigative behaviour was accompanied by a decrease in walkover episodes (genotype: F_2, 29_ = 7.403, p<0.01) and walkover time (genotype: F_2, 29_ = 4.33, p<0.01) compared to control mice, indicating a reduction in social dominance-related behaviour (Figure 7H and I).

## Discussion

The results described above implicate Sema6A in the control of neurodevelopmental processes across the brain, including novel and important functions in the developing hippocampus and cortex. Convergent findings from physiological, behavioural and pharmacological experiments also suggest that the neurodevelopmental defects observed in *Sema6A* mutants result in endophenotypes that parallel some aspects of psychiatric disorders.

Our findings extend the functions of Sema6A in cell migration to various areas of the brain, including the hippocampus, piriform cortex and neocortex. They also extend its role in axon guidance to the lateral olfactory tract, anterior commissure and fornix. A new trend that is apparent is the role of Sema6A in restricting neuronal cell bodies from areas of neuropil and in the organisation of distinct tracts within neuropil. One interesting question is how this disorganisation or misrouting of axons affects connectivity. For the axons of the posterior limb of the anterior commissure it is clear that they are misrouted into the ipsilateral hypothalamus and septum but we have not ascertained whether they make functional connections there; they clearly do not make functional connections with their normal targets in the contralateral hemisphere. Analysis of the connectivity of misplaced cells in the cortex and hippocampus will require electrophysiology at the microcircuit level (e.g., [52]).

Our focus in this paper is not on the primary functions of Sema6A in these processes, however, nor on using these phenotypes to understand how Sema6A works in neural development. These questions have previously been addressed in many different areas of the developing nervous system [30], [31], [32], [33], [36], [38], [39], [40]. Our interests here are in cataloguing the spectrum of anatomical defects in these mutants and examining their combined effects on the functioning of the nervous system, as assessed at the physiological and behavioural levels, in order to explore the possible relevance of such effects to the etiology of neurodevelopmental disorders in humans. It is important to note that Sema6A may also have additional functions in adults, in synaptic plasticity or related processes, for example, which could also contribute to the physiological and behavioural phenotypes we observe.

We consider the phenotypes observed in *Sema6A* mutants in the context of what is known of the neuropathology, pathophysiology and pharmacology of psychiatric disorders, especially SZ and ASD. Given that each of these diagnostic categories most likely represents a collection of disorders with diverse etiologies, these comparisons are necessarily general. We also compare the defects observed here to those seen in mouse mutants of validated psychiatric disease genes or non-genetic models of psychiatric disorders.

### Comparison with the pathology of psychiatric disorders

Neuropathological studies of *post mortem* brains of SZ patients provide evidence for variable and subtle cytoarchitectural disturbances in cell positioning, packing, density and size in various parts of the brain, most notably the prefrontal cortex, hippocampal formation [4], thalamus [53] and cerebellum [54], [55]. A general comparison with the phenotypes observed in *Sema6A* mutants suggests at least a qualitative similarity, with small numbers of misplaced cells in various brain regions. For example, misplaced clusters of neocortical layer 2 cells and ectopic neurons in the white matter have been repeatedly observed in *post mortem* brains of SZ patients [56], [57].

In ASD, neuropathological studies have similarly highlighted a number of aberrations in the cellular organization of the cortex [11]. These include irregular lamination, ectopic neurons in layer 1 (the molecular layer) and in the white matter and irregular clumping of neurons in the grey matter [58], [59], [60], implicating a primary defect in cortical cell migration. Ectopic granule cells have also been observed in the molecular layer of the dentate gyrus and of the cerebellum [60], both of which we also observe in *Sema6A* mutants (this study and [39]). It should be noted that for both SZ and ASD, no single phenotype is seen in all cases, presumably reflecting the underlying heterogeneity of genetic causes [20], [22].

Despite the expected phenotypic heterogeneity, diffusion-weighted neuroimaging studies have yielded broadly consistent findings of dysconnectivity in limbic, thalamocortical and intracortical tracts in SZ and BD [5], [6], [7], [8], [9], [61], [62], [63], some of which parallel those in *Sema6A* mutants remarkably closely (this study and [40]). These include a well-replicated disorganization and reduction in the cross-sectional area of the fornix [64], [65], [66], which has been quantitatively correlated with impairment on several memory tasks [67], [68], [69]. Similar analyses in ASD also reveal widespread alterations in structural connectivity [70].

Pathology in SZ and ASD also extends to motor and sensory systems, consistent with symptoms in these domains [71]. Implicated motor systems include the cerebellum [13], [54], [55] and corticospinal tract [70], [72], where we have previously described defects in *Sema6A* mutants [30], [39]. The abnormalities in gait that we report here may reflect disrupted cellular organisation in the cerebellum [73]. Deficits in early visual processing in SZ patients [74] are consistent with well-replicated observations of reduced connectivity between lateral geniculate nucleus and primary visual cortex [6], [61], [75], [76] and a reduction in the size of primary visual cortex [77], both of which are observed in adult *Sema6A* mutants [40].

### Behavioural phenotypes and alteration in cortical physiology

*Sema6A* mutants display robust hyper-exploratory behaviour and hyperlocomotion, both of which are seen across multiple test environments. Detailed analysis of the *ethogram*, which evaluates the naturalistic behavioural repertoire, shows that this hyperactivity is not accompanied by an increase in stereotypic behaviours but reflects both increased locomotion and increased exploration, thus implicating motivational as well as motor systems.

Hyperlocomotion is arguably the most consistently observed phenotype across many genetic, surgical or pharmacological preclinical models of SZ [27], [51], [78], [79], [80], [81], including mutants in *DISC1* [82], [83], [84], *Nrg1* [85] and *NPAS3* [86], ventral hippocampal lesion models [87] and chronic or acute treatment with NMDA-receptor blockers [88], [89] or amphetamine [78], [79], [90], [91], [92], [93], both of which can induce psychotic states in humans. Importantly, the hyperlocomotion in *Sema6A* mutants, as in these other models, is reversible by treatment with antipsychotics, demonstrating the predictive validity of this phenotype as a model for psychosis and differentiating it from hyperlocomotion induced by drugs such as nicotine.

Most of these animal models are also characterised by a hyperdopaminergic state [78], [90], [91], [94], [95]. This is highly congruent with findings in humans where a psychotic state is associated with an increase in dopamine release and/or responsiveness [96]. Alterations in dopamine signalling may thus represent a final common pathway to psychosis, one which can be induced through a wide variety of insults [94], [97], [98].

The amphetamine sensitisation model, which directly alters dopaminergic signalling and which is well accepted as a model of psychosis [79], [93], is also characterised by an EEG phenotype that is highly similar to that observed here in *Sema6A* mutants – namely, a selective increase in alpha power [99]. Similar results have been reported in a number of other animal models with a hyperdopaminergic state [100], [101], [102].

These comparisons thus suggest that both the hyperactivity and the EEG phenotype in *Sema6A* mutants may be associated with alterations in dopaminergic signaling. The fact that both are reversible by antipsychotics that target D1 and D2 receptors is highly consistent with such a hypothesis. We have not, however, observed significant differences in *Sema6A* mutants in tonic dopamine levels or in the abundance of D2 receptors in striatum or prefrontal cortex (Supporting Information S1). Nevertheless, it remains possible that there is increased acute release of or response to dopamine under certain conditions, as seen in *DISC1* mutants, for example [103] or altered levels of specific isoforms of D2 receptors that we have not assayed [98]. We note that early disruptions to hippocampal circuitry are known to induce circuit-level homeostatic mechanisms altering dopaminergic tone or responsiveness in the prefrontal cortex and striatum [87], [104]. Whether the defects we observe in hippocampal circuitry in *Sema6A* mutant mice could be having such an effect will require further investigation. It is also possible that these phenotypes are caused by disturbances in other systems that can be compensated for by altering dopaminergic signalling.

### Cognitive phenotypes

*Sema6A* mutants also display some evidence for cognitive phenotypes, including deficits in working memory and novel object recognition [26], [79]. Impairment in spatial working memory is considered a core cognitive deficit in SZ [105], and is observed in many relevant animal models [79]. The fact that the working memory defect is only evident in males indicates that this defect is not simply a result of increased locomotor activity, which is seen in both sexes in this and other tests. However, further examination of working memory processes using more complex maze task variants are required to characterize the nature and severity of this putative deficit.

Defects in novel object recognition can be induced by NMDA-R blockers or by methamphetamine sensitisation [79] and NOR is considered a valid animal model of human declarative memory [106], which is frequently impaired in SZ. A deficit in this test could alternatively arise due to altered salience. This would be consistent with the increased exploration of both known and novel objects that we observe in *Sema6A* mutants. Though speculative, the latter explanation would also fit with a model of hyperdopaminergia [79], which is linked to salience-related effects in humans with psychosis [107], [108].

*Sema6A* mutants show an anxiolytic profile across two measures of anxiety-related behaviour, which cannot be explained with reference to the observed hyper-exploratory phenotype. Mice mutant for the *Sema3E* gene also demonstrate reduced anxiety in the elevated plus maze, which may be attributable to a defect in the fornix of these animals [109], suggesting a similar possible explanation in *Sema6A* mutants. The effect on anxiety in *Sema6A* mutants is particularly interesting in light of the strong association of *PLXNA2* variants with anxiety in humans [110].

### Links to the etiology of psychiatric disorders in humans

The phenotypic spectrum described here suggests that mutations in *SEMA6A* in humans would likely result in a psychiatric presentation of some sort. *SEMA6B*, *PLXNA2* and *PLXNA4* are similarly implicated by virtue of their biochemical and genetic interactions and overlapping spectrum of anatomical phenotypes when disrupted [30], [31], [32], [33], [35], [36], [37]. Though no discrete mutation in any of these genes has yet been discovered in human patients, there is convergent evidence implicating either their chromosomal regions or common polymorphisms in the genes themselves.

*SEMA6A* falls in one of the best-replicated linkage peaks for SZ, on 5q23, first discovered in Irish families [111], [112]. A study using dense mapping in extended Swedish pedigrees identified a haplotype inherited identical by descent in the 5q23.1 region, segregating in individuals diagnosed with SZ [113]. This haplotype is restricted to a 6.8 Mbp region, which encompasses sixteen genes, one of which is *SEMA6A*. Rare deletions of the 5q23 region, which include the *SEMA6A* locus, have been found in patients with mental retardation and SZ [114], [115], [116].

*PLXNA2* is also located in a tightly replicated linkage peak for SZ and for periodic catatonia at 1q32.2 [117], [118], [119]. In addition, both *SEMA6B* and *PLXNA4* fall near the maxima of very tightly defined linkage peaks for autism, derived from multiplex pedigrees, on 19p13.3 [120] and 7q32.2 [121], respectively. Given the phenotypes described above, these genes are plausible candidates to underlie these linkage findings.

*PLXNA2* also emerged as the top candidate from a whole-genome case-control association analysis of SZ in a population of European descent [41]. This result was replicated in several independent samples [41], [42], though not in all populations [41], [122], [123]. Nevertheless, a comprehensive meta-analysis ranked *PLXNA2* among the top SZ risk-associated genes [43]. *SEMA6A* has been recently associated with ASD, in a novel genome-wide association analysis method [44].

We have replicated evidence for an association of *PLXNA2* variants and SZ risk in a case-control study from the Irish population, though only at a statistically suggestive level (Supporting Information S1). We also found suggestive evidence for an association with variants in *SEMA6A* and *SEMA6B* and a stronger epistatic interaction between the associated variants in *PLXNA2* and *SEMA6B* (Supporting Information S1). Biological epistatic interactions between these genes have been documented in the control of hippocampal connectivity in mice [33], highlighting the plausibility of such an interaction. Given the effect sizes and p-values, the associations we observe may be false positives. If real, they could represent either a small effect of a common variant on risk in individuals or a larger effect of a linked, but more highly penetrant mutation that is much rarer in the population [124]. Such mutations would be difficult to detect in large-scale genome-wide association studies that combine samples across populations. Whether disease-causing variants in these genes exist in human populations will thus require further investigation.

In summary, we present evidence that mutation of the *Sema6A* gene results in widespread defects in cellular organisation and axonal projections, some of which resemble those reported in ASD and SZ. This demonstrates that this kind of neuropathology can arise directly from mutations in a gene controlling cell migration and axon guidance. We also show that these mutants display a behavioural and physiological profile with similarities to accepted animal models of SZ, particularly the positive symptoms of psychosis. We do not propose that these mutants provide a model for SZ or ASD as a whole. These disorders are so heterogeneous that it is unlikely that any single mutant mouse or even any individual human patient could model the entire spectrum of either of these diseases. However, *Sema6A* mutants may provide an informative model to investigate how defects in neurodevelopmental processes, can, through either primary or reactive mechanisms, cause neuronal network and circuit dysfunction and ultimately result in a specific profile of behavioural deficits of relevance to psychopathology in humans. Given these findings, *SEMA6A* and the genes encoding interacting proteins become interesting candidates to explain some of the linkage findings for SZ, BD and ASD in their respective chromosomal loci.

## Materials and Methods

### Animal use

All animal procedures were performed in accordance with the European Communities Council Directive (86/609/EEC). Those carried out at Trinity College were reviewed and approved by the Trinity College BioResources Committee under license B100/3527 (licence holder: KJM) from the Department of Health, Dublin, Ireland. Those carried out at the Royal College of Surgeons in Ireland were approved by the Research Ethics Committee of the Royal College of Surgeons in Ireland REC 092 REC205A and were conducted under licenses B100/759 (licence holder: JLW), B100/962 (licence holder: CO'T) and B100/3248 (licence holder: MD) from the Department of Health and Children.

### Genotyping

Genotyping of *Sema6A* mice was carried out as previously described [30].

### Histology

PLAP staining was carried out as previously described [38], as were tracings with lipophilic DiI and immunhistochemistry [30]. The following antibodies were used: rat anti-L1 (Chemicon), mouse anti-NeuN (Chemicon), goat anti-Neuropilin-1 (R&D Systems), rabbit anti-Prox1 (Chemicon).

### Electroencephalography

EEG recordings and analyses were carried out essentially as described [125]. There was no significant difference in weights between *Sema6A* homozygotes and heterozygotes. Female mice were anesthetized using isoflurane (5% induction, 1–2% mainenence in O_2_). Mice were placed in a stereotaxic frame (David Kopf Instruments, CA, USA) and body temperature maintained at 37±0.5°C by means of a homeostatic blanket and heat lamp (Harvard Apparatus, MA, USA). Following a midline incision Bregma was located and three partial burr holes drilled bi-temporally overlying the hippocampi and a third on the midline over the frontal cortex. Cortical EEG was recorded by means of skull-mounted recording electrodes (Plastics One Inc, VA, USA) fixed with dental cement. Electrodes were connected to a Comet EEG acquisition system (Grass-Telefactor, USA) and recordings commenced. Right and left side EEG was recorded for *Sema6A* heterozygous and homozygous mice for 20 minutes, during which 5-second segments were selected and spectral analysis performed for left and right sides. Baseline EEG was recorded for 20 min followed by injection of clozapine (0.25 mg/kg i.p.) and continued for 40 min post-injection. EEG spectral analysis was divided into standard frequency bands (beta, theta, alpha, delta) and the relative input into total spectrum of each band was calculated automatically by an in-built feature of the analysis software (Twin, Grass-Telefactor, USA) for 5 s epochs. For technical reasons, it was not possible to estimate gamma frequency power from these recordings. Baseline values were calculated in the 5 min pre-injection. The signal was analysed 25 min. post injection, since altered clozapine-induced behaviour has been shown to emerge at this time. All data are expressed as mean ± SEM.

### Behavioural Analyses

#### Footprint Test

The footprint test was used to assess gait [126]. Black and red paint was daubed on the hind- and forefeet, respectively, of each mouse. Animals were then permitted to walk along a brightly lit 34 cm-long, 14 cm-wide runway (10-cm-high perspex walls) into a darkened box lined with sample of bedding from mouse's home cage. A sheet of white paper lined the floor of the runway. The following parameters were measured (cm): Stride length – average distance between each stride; hind-base width – average distance between left and right hind footprints; front-base width - average distance between left and right front footprints; left-right overlap – difference from left or right front footprint/hind footprint overlap.

#### Wire-hang suspension

Grip strength was assessed in the wire-hang suspension task [127]. Each mouse was placed on a wire net (30×35 cm) with plastic borders along its sides. The wire netting was given a quick shake and turned upside down (approx. 30 cm above a worktop). The amount of time that each mouse held onto the wire was recorded up to a maximum of 300 s.

#### Elevated-plus maze

Anxiety-related behaviour was assessed using the elevated plus maze test as described [128]. Two opposing arms were surrounded by cream-coloured chipboard walls (12 cm high, closed arms); the other two walls were devoid of walls (open arms). The apparatus was elevated 25 cm above ground level. The test was conducted under dim lighting conditions. At the start of test, the mouse was placed in the centre of the maze, facing one of the open arms. Number of entries into each arm, as well as cumulative time spent in open and closed arms was recorded during a 5 min session. Entry into an open arm was defined as all four paws being placed in that arm. The maze was cleaned between trials with 3% Virkon™ (Antec International, USA). The start of each trial coincided with the mouse's first arm entry.

#### Marble-burying task

In the marble-burying task, mice were individually placed in clear perspex boxes (33×15×13 cm), containing 25 glass marbles (diameter −1.5 cm) evenly spaced on sawdust (depth-5.2 cm). Mice were not allowed access to food and water. Marble-burying behavior was defined as the number of marbles buried at least two-thirds deep in the sawdust within 30 min.

#### Monitoring of activity in a novel environment

Locomotor activity was assessed in the open field. Each mouse was placed individually in the center of an open field apparatus (ENV-510; 27.9×27.9 cm; Med Associates, St. Albans, VT). Total ambulatory counts, distance traveled (cm), and vertical activity were recorded. Data were collected over a 60-min period.

#### Reversal of hyperactivity following antipsychotic administration

Activity was measured in mice pre-treated with the atypical antipsychotic clozapine (0.25, 0.5, 1.0 mg/kg) or the typical antipsychotic haloperidol (0.5 mg/kg). Animals were brought from the holding facility into the experimental room. They were then weighed and injected s.c. with vehicle or drug and placed back in their home cages. 20–25 minutes later, each mouse was placed individually in the open field apparatus (ENV-510; 27.9×27.9 cm; Med Associates, St. Albans, VT) and several indices of activity (see above) were recorded over a 60-min period.

Clozapine (Sigma/RBI, St. Louis, MO) was dissolved in 50 µl 0.1 M HCL and diluted in distilled water. Haloperidol (Sigma, St. Louis, MO) was dissolved in glacial acetic acid and diluted in distilled water. Vehicle consisted of 50 µl 0.1 M HCL – distilled water. All solutions were prepared fresh daily and injected in a volume of 4 ml/kg.

#### MK-801 induced hyperactivity

Animals were brought from the holding facility into the experimental room. They were then weighed and injected s.c. with vehicle or MK-801 (0.2 mg/kg) and returned to their home cages. 20–25 minutes later, each mouse was placed individually in the open field apparatus (ENV-510; 27.9×27.9 cm; Med Associates, St. Albans, VT) and several indices of activity (see above) were recorded over a 60-min period. MK-801 (Sigma/RBI, St. Louis, MO) was dissolved in distilled water. Vehicle consisted of distilled water. All solutions were prepared fresh daily and injected in a volume of 4 ml/kg.

#### Social interaction in a novel environment

In the test of social interaction in a novel environment, mice were individually paired with an unfamiliar age-, weight- and sex-matched C57BL6 mouse in a novel observation chamber (28×28×16 cm) for a 10 min observation period. The chamber was constructed of clear perspex and clean bedding material was placed on the chamber floor prior to each test. Between each test, the chamber floor and walls were cleaned with 3% Virkon™ (Antec International, USA). Both the test mouse and the unfamiliar C57BL6 conspecific were placed in the chamber simultaneously and this placement defined the start of the trial. For each animal, the test was captured and recorded using a digital camcorder mounted above the chamber at ceiling level. The experimenter was blind to genotype at time of test and during the subsequent coding of behaviours. All social and nonsocial exploratory behaviours were later coded using the Observer ® (Noldus Inc., The Netherlands) video analysis software. Nine behaviours were coded and organised into the following behavioural domains: social investigation (anogenital sniffing – sniffing of anogenital region of conspecific), social dominance (walkover– the mouse places its front paws on the head or back of conspecific; aggressive following – the test mouse rapidly follows the conspecific from behind, forcing it to retreat), agonistic behaviours (pinning – the mouse pins the conspecific to the floor; clawing), nonsocial exploration (rearing to wall; rearing free – the mouse is upright with front paws raised; sifting – the mouse sifts through the bedding). All behaviours coded were those initiated by the test mouse.

#### Spontaneous alternation memory

The continuous variant of the Y-maze spontaneous alternation procedure was assessed during one 10 min session. The Y-maze apparatus consisted of three identical arms (40×12.5×40 cm). Without prior habituation, each test mouse was placed at the centre of the Y-maze and allowed to move freely throughout the maze for a single 10 min period. Rodents possess a natural preference to explore areas previously un-explored; if a mouse has explored one arm of the Y-maze, it is not expected to enter the same arm during its next phase of exploration, but to enter one of the two alternate arms; this test has been suggested to measure several aspects of spatial working memory [129]. A video camera, mounted centrally above the Y-maze, recorded each session and allowed alternation to be analysed using video tracking software (Ethovision®, Noldus Inc., the Netherlands). Spontaneous alternation was defined as successive entries into the three arms, in overlapping triplet sets. It is expressed as a percentage and refers to the ratio of arm choices differing from the previous two choices to the total number of arm entries: percent alternation = ((number of alternations/total number of arm entries) – 2)×100 [129]. The experimenter was blind to genotype at time of test.

#### Novel object recognition

Recognition memory was assessed using the novel object recognition paradigm with short (1 hr) or long (4 h) retention times, respectively. The apparatus consisted of an open arena (29×29×20 cm) covered by a layer of bedding (depth 2.5 mm) surrounded by a black cardboard wall. The objects (A, B, C) were two golf balls (diameter 5 cm) with a dimpled surface texture, a blue and a green opaque hollow plastic disc (diameter 4.5 cm, height 2.5 cm) with two small holes in surface, and two pink plastic Lego® bricks (dimensions 5×3.5×2.5 cm)) with 8 protrusions. The experiment was conducted under standard room lighting.

Habituation phase. Twenty four hours prior to testing, mice were placed in the arena and allowed to explore freely for two consecutive sessions of thirty minutes each, with an inter-session interval of 15 min; mice were returned to their home cage between habituation sessions. The arena was wiped clean using 5% Virkon and bedding replaced, both between each session for a given animal and between each experimental animal.

Sample phase. For testing, mice were first allowed to explore the arena for two further sessions of 30 min separated by an inter-session interval of 15 min. Then, two identical objects, A1 and A2, were placed at the rear left and right corners of the arena, 5 cm from both walls to facilitate exploration around each object. Each test mouse was placed at the midpoint of the wall opposite to the sample objects, with nose facing the wall and hence away from objects so as to remove any unintentional bias and allowed to explore the arena for 5 min. Time spent interacting with each object was recorded manually. At the conclusion of this 5 min period, the test mouse was returned to its home cage, the arena cleared of bedding and both arena and objects wiped down to remove odour traces.

Test Phase 1. Following an inter-trial interval of 1 h, the test mouse was placed in the arena now containing familiar object A1 and novel object B1 for 5 min. Time spent interacting with each object was recorded manually for 5 min.

Test Phase 2. Following an inter-trial interval of 4 h (i.e. the period between completing Test Phase 1 and initiating Test Phase 2), the test mouse was placed in the arena now containing familiar object B1 and novel object C1. Time spent interacting with each object was recorded manually for 5 min. The sequence of object presentation was counterbalanced between animals (i.e. which object served as sample or trial object). Similarly, the position of novel and familiar objects was counterbalanced between trials (i.e. novel or familiar object placed in left or right corner of arena). Time spent interacting with each object was determined as nose or whiskers touching the object or directed towards it within <1 cm, or touching the object with a paw. Accidental contact with object (i.e. bumping or using the object as a platform to explore the arena) was not counted as interaction with the object. The experimenter was blind to genotype at time of test and during the subsequent coding of behaviours.

#### Statistical Analyses

Analysis of behavioural data followed procedures similar to those described previously [50], using between- or within-subjects analysis of variance (ANOVA), as appropriate, with main factors of genotype, sex, and/or drug treatment; *post-hoc* comparisons were carried out using independent or paired *t*-tests, corrected for multiple comparisons. Gait measures were analysed in terms of total length for each measure; exploration in a novel environment was analyzed in terms of activity counts and distance travelled over 1–6 h of habituation; exploration in a novel environment following prep-treatment with an antipsychotic or MK-801 was analyzed in terms of activity counts; spontaneous alternation was analyzed in terms of percentage alternation and number of arm entries; novel object recognition was analyzed in terms of time exploring the novel object during the sample phase, test phase 1, and test phase 2; social interaction was analyzed in terms of total number of episodes and total duration of each behaviour; the EPM was analyzed in terms of absolute or percentage time spent in, and number of entries into, each arm; marble burying was analysed in terms of total number of marbles left uncovered. For the above phenotypic measures, we hypothesized that genotype would be modulated by sex [50]; thus, where appropriate, separate *t*-test analyses were also conducted within male and female groups. All statistical analyses were carried out using the SPSS software package (Version 14, SPSS, Chicago, IL, USA).

### High-performance liquid chromatography analysis

Tissue constituting three brain areas (frontal cortex, striatum, hippocampus) was removed from each of 16 mice (3 Sema6A^+/−^ male, 4 Sema6A^−/−^ male, 4 Sema6A^+/−^ female, 5 Sema6A^−/−^ female). All were 10 weeks old, except for 2 female homozygous mutants, which were 15 weeks old. Tissue was weighed and put into 500 µl HPLC buffer (buffer was previously run through HPLC column to get baseline standards), sonicated to homogenise, centrifuged 10 min at 1000 g and stored at −70°C until use. 100 µl of each sample was analysed by HPLC to ascertain levels of dopamine and serotonin and their metabolites 3,4-Dihydroxyphenylacetic acid (DOPAC), homovanillic acid (HVA) and 5-Hydroxyindoleacetic acid (5-HIAA).

## Supporting Information

Supporting Information S1 This file contains detailed information on cell positioning defects of anterior commissure-projecting neurons and concentrations of neuromodulators in various brain regions in *Sema6A* mutant animals. It also describes methods and results of association analyses of variants in *SEMA6A*, *SEMA6B*, *PLXNA2* and *PLXNA4* with schizophrenia in a sample from the Irish population.
(DOC)
