# Supplementary material for: Mutation of Semaphorin-6A Disrupts Limbic and Cortical Connectivity and Models Neurodevelopmental Psychopathology
Source: PLoS One. 2011 Nov 21;6(11):e26488. doi: 10.1371/journal.pone.0026488 (PMC3221675; doi:10.1371/journal.pone.0026488)
Supplement: Supporting Information S1 — This file contains detailed information on cell positioning defects of anterior commissure-projecting neurons and concentrations of neuromodulators in various brain regions in Sema6A mutant animals. It also describes methods and results of association analyses of variants in SEMA6A, SEMA6B, PLXNA2 and PLXNA4 with schizophrenia in a sample from the Irish population. (DOC) [file pone.0026488.s001.doc]

**Supporting Information S1**

**Cell positioning defects in prefrontal cortex**

To retrogradely label the cell bodies that give rise to the pAC axons, we performed dye-tracing experiments. Crystals of DiI were placed either at the location of the pAC at the midline of transected brains from heterozygous animals (Figure 2A and B) or at the ventral surface of the hypothalamus, to where many of the misrouted axons project, in homozygous mutants (n=4, Figure 2C and D). In heterozygotes, these dye placements retrogradely labelled cells in the insular cortex from very rostral levels (Figure 2A1 and B1, arrowheads) to levels well caudal to the pAC (Figure 2A5 and 2B5). In contrast, in homozygous mutants, retrograde tracing of the misrouted axons labelled virtually no cells within the insular cortex, but did label cells in a more ventral position, in the dorsal part of the piriform cortex (Figure 2C1-3 and D1-3). Only very few labelled cells were found caudal to the level of the pAC (Figure 2C4-5 and D4-5). Notably, tracings in heterozygous mice did not label cells in the dorsal piriform cortex, thereby excluding the possibility that the difference in the position of cells that give rise to the pAC might be due to tracing of only a subset of pAC axons. The cells that normally project axons across the pAC are thus defective in both axonal projections and cell body positioning.

**
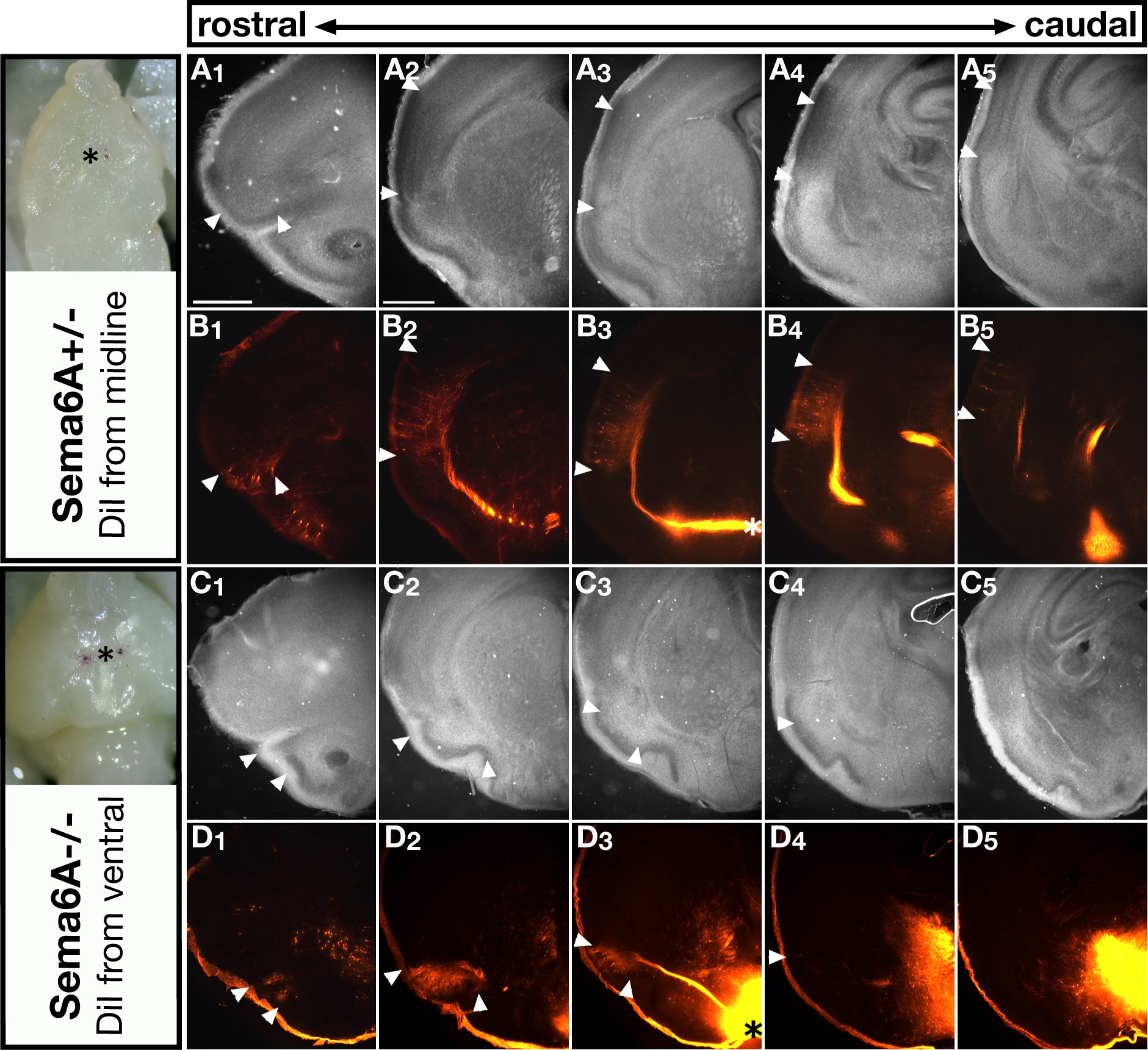
**

**Supplementary Figure S1: Cellular disorganisation in piriform and insular cortex.** DiI tracing of the pAC (and aAC) axons from the midline of transected brains of newborn *Sema6A^+/-^* mice (A, B) and of misrouted pAC axons from the ventral surface of *Sema6A^-/-^* mice (C, D). (A, C) show phase contrast views of the same sections shown in (B, D). In *Sema6A^+/-^* mice, back-labeled cells were located in the insular cortex and ventral neocortex from very rostral to levels far caudal to the level of AC midline crossing (arrowheads in A, B). In contrast, cells that give rise to the (misrouted) pAC of *Sema6A^-/-^* mice are located in the dorsal piriform cortex from very rostral levels but not far further caudal than the level of AC midline crossing (arrowheads in C, D). Notably, AC tracing in *Sema6A^+/-^* mice did not back-label cells within the dorsal piriform cortex. Scale bar in (A2) is for (A-D2-5), and in (A1) is for (A-D1): 500 µm.

**High-pressure liquid chromatography of neuromodulators**

We performed HPLC analyses to ascertain levels of dopamine, serotonin and their major metabolites, DOPAC, HVA and 5-HIAA, in striatum, hippocampus and frontal cortex of homozygous mutant and heterozygous mutant Sema6A animals, as described in Materials and Methods. Ten-week old male and female mice were analysed for each genotype. No significant differences for genotype were observed (Supplementary Table 1).

**Supplementary Table 1. Neuromodulator concentrations in *Sema6A* mutants.** Mean concentrations for each substance are given in ng/g for heterozygous (Het) or homozygous (Hom) Sema6A mutant males (M), females (F) or both sexes combined (M+F) across each of three dissected brain regions. T-tests did not reveal any significant differences between genotypes for any of these comparisons, with one exception (5-HT concentration in male hippocampus, p = 0.03), which would not survive correction for multiple testing.

|  |  |  |  | **Frontal Cortex** |  |  |  |  | t-tests |  |
| --- | --- | --- | --- | --- | --- | --- | --- | --- | --- | --- |
|  |  | **Het M** | **Hom M** | **Het F** | **Hom F** | **Het (M+F)** | **Hom (M+F)** | Het x Mut (M) | Het x Mut (F) | Het x Mut (M+F) |
| **Mean** | DA | **934.373754** | **167.493736** | **218.1196543** | **193.2272368** | **536.4548097** | **184.6494032** | 0.493446073 | 0.581837969 | 0.369574807 |
| *Standard Error* |  | *679.1276518* | *3.07181479* | *32.1962058* | *26.12799004* | *304.9441803* | *17.41964528* |  |  |  |
|  | DOPAC | **310.5447904** | **191.6215465** | **308.5069464** | **197.8877796** | **309.5258684** | **195.7990352** | 0.51330089 | 0.23109746 | 0.138454651 |
|  |  | *96.4687824* | *78.94033484* | *59.28337251* | *42.15518793* | *60.44314673* | *36.54759548* |  |  |  |
|  | 5HT | **1530.036936** | **1482.268755** | **1769.344171** | **1491.929577** | **1662.9854** | **1487.789225** | 0.789110261 | 0.061072608 | 0.11913861 |
|  |  | *127.8006389* | *90.94483981* | *103.8770551* | *45.79630243* | *86.52769375* | *42.24458149* |  |  |  |
|  | 5HIAA | **824.152514** | **687.5001831** | **803.5261155** | **783.4315424** | **812.6934037** | **742.3181027** | 0.332500156 | 0.830353594 | 0.342556696 |
|  |  | *101.4794024* | *50.94269757* | *67.66110826* | *54.33903152* | *54.7827504* | *39.87480031* |  |  |  |
|  | HVA | **646.7025496** | **583.8844821** | **480.6925569** | **401.7671224** | **554.4747759** | **479.8174194** | 0.72505538 | 0.216945353 | 0.40403634 |
|  |  | *113.1065799* | *122.9820839* | *46.99366024* | *26.55480206* | *59.9685324* | *60.95796833* |  |  |  |
|  |  |  |  |  |  |  |  |  |  |  |
|  |  |  |  | **Striatum** |  |  |  |  | t-tests |  |
|  |  | **Het M** | **Hom M** | **Het F** | **Hom F** | **Het (M+F)** | **Hom (M+F)** | Het x Mut (M) | Het x Mut (F) | Het x Mut (M+F) |
| **Mean** | DA | **167018.5963** | **222555.1495** | **237558.5722** | **232766.9347** | **206207.4718** | **228390.4553** | 0.497696342 | 0.857615696 | 0.525280789 |
| *Standard Error* |  | *54963.93001* | *46991.79599* | *17982.806* | *17889.87025* | *27329.66408* | *20277.07449* |  |  |  |
|  | DOPAC | **29132.95075** | **35037.67812** | **31280.0161** | **37470.73384** | **30325.76483** | **36427.99568** | 0.65381096 | 0.231598292 | 0.253288833 |
|  |  | *9882.235547* | *4903.939111* | *3547.654599* | *2824.988824* | *4462.597984* | *2440.758154* |  |  |  |
|  | 5HT | **20635.50167** | **16720.83797** | **16315.40022** | **15302.97399** | **18235.44531** | **15910.62998** | 0.396005288 | 0.641642549 | 0.269080545 |
|  |  | *3554.148401* | *397.4866914* | *1391.791979* | *1546.352785* | *1794.278315* | *887.5964254* |  |  |  |
|  | 5HIAA | **19629.22991** | **16723.72851** | **14544.38358** | **17427.79906** | **16804.31528** | **17126.05454** | 0.505163619 | 0.18458578 | 0.871625158 |
|  |  | *3385.301507* | *709.755608* | *1519.118071* | *1063.022004* | *1830.052432* | *644.2512307* |  |  |  |
|  | HVA | **32439.91417** | **45687.05027** | **35767.80184** | **41196.79164** | **34288.74065** | **43121.18819** | 0.283339571 | 0.272220777 | 0.088445339 |
|  |  | *8224.330031* | *6193.931524* | *3492.77037* | *2563.662586* | *3873.476221* | *2860.324946* |  |  |  |
|  |  |  |  |  |  |  |  |  |  |  |
|  |  |  |  | **Hippocampus** |  |  |  |  | t-tests |  |
|  |  | **Het M** | **Hom M** | **Het F** | **Hom F** | **Het (M+F)** | **Hom (M+F)** | Het x Mut (M) | Het x Mut (F) | Het x Mut (M+F) |
| **Mean** | DA | **211.1042283** | **138.8188466** | **105.9232413** | **169.545733** | **192.3138933** | **165.9921093** | 0.302425179 | 0.89901614 | 0.258889719 |
| *Standard Error* |  | *29.29330823* | *39.76926987* | *53.11870001* | *16.56401008* | *19.20999773* | *10.86878442* |  |  |  |
|  | 5HT | **1687.379757** | **1740.229553** | **1478.674999** | **1614.700507** | **1716.740755** | **1556.403861** | **0.030597511** | 0.351997344 | 0.056005403 |
|  |  | *56.66943151* | *104.502202* | *23.60800506* | *48.48310408* | *60.456388* | *38.8129481* |  |  |  |
|  | 5HIAA | **1600.571729** | **1579.625915** | **1501.562013** | **1915.787277** | **1588.935166** | **1738.262163** | 0.449578266 | 0.095925368 | 0.310764826 |
|  |  | *94.60659772* | *74.95477779* | *54.14402876* | *173.9409529* | *55.37049748* | *126.7540235* |  |  |  |
|  | HVA | **271.786665** | **260.0353333** | **284.9430022** | **248.4487255** | **265.2581474** | **308.1039848** | 0.783529182 | 0.892103737 | 0.208869503 |
|  |  | *36.43446298* | *19.81144668* | *16.94843997* | *90.23391214* | *18.29024657* | *26.04203798* |  |  |  |

## Human genetic analyses

To assess whether genetic variation in *SEMA6A* or in the interacting genes *SEMA6B*, *PLXNA2* and *PLXNA4*, might contribute to the etiology of SZ we performed case-control association analyses. The sample comprised 375 cases and 812 controls from the Republic of Ireland. Among 72 informative SNPs genotyped, seven SNPs across 3 of the 4 genes showed suggestive evidence of association with SZ (Supplementary Table 2).

*SEMA6A*: Three SNPs, rs258015, rs258016 and rs41099, were associated with SZ (p < 0.05). All 3 SNPs are located in a small 3kb region of high LD (*r^2^* > 0.5), with rs258015 and rs258016 both positioned in a conserved region of intron 3 of the gene. Haplotype analysis in the region of these 3 SNPs produced significant results but the association signal was not stronger than that identified for each SNP individually.

*SEMA6B*: The two associated SNPs in this gene, rs10409783 and rs10422881, are in high LD (*r^2^* > 0.5). rs10422881 is located in intron 3, 31bp from the start of exon 4, and rs10409783 is located in intron 5 of the gene. Haplotype analysis combining these 2 SNPs detected a stronger association than for either SNP individually (*omnibus test,* p = 0.014; *f*(A-A) in cases = 0.268, *f*(A-A) in controls = 0.218, p = 0.009).

*PLXNA2*: Two SNPs, rs2281913 and rs7540179, were associated with SZ (p < 0.05). For each SNP, the association was identified with the genotype test and the signal was due to the lower frequency of heterozygotes in cases compared to controls. A third SNP at this locus, rs6656034, showed a similar trend-level genotype association (p = 0.059; also included in Table 1). These 3 SNPs are not in substantive LD with each other (*r^2^* < 0.5). Haplotype analysis detected association with the rs7540179-rs2498028-rs10863694 combination (*omnibus test,* p = 0.014) due to the A-A-A haplotype: (*f*(A-A-A) in cases = 0.085, *f*(A-A-A) in controls = 0.126, p = 0.004). The 3 single associated SNPs and the associated haplotype are located in a ~46kb region that spans exons 10-21 and contains 4 SNPs previously associated with SZ . These 4 SNPs did not individually show evidence of association in this study but rs2498028 is part of the associated haplotype.

*PLXNA4*: Single marker or haplotype analysis did not identify association between this gene and SZ.

None of the significant single SNP or haplotype association results reported here survive correction for multiple testing using permutations; these results alone should therefore be interpreted as suggestive. However, epistasis analysis revealed a more statistically robust association.

|  |  |  |  |  |  |  |  |  |  |  |  |  |
| --- | --- | --- | --- | --- | --- | --- | --- | --- | --- | --- | --- | --- |
| **Gene** | **rs Number** | **Location** | **Alleles^a^** |  | **Allele counts in Cases** | **Allele counts in Controls** | ***P Value***  ***(Sim. P)^b^*** | **OR (95% CI)** |  | **Genotype counts in Cases** | **Genotype counts in Controls** | ***P Value***  ***(Sim. P)^b^*** |
| PLXNA2 | rs2281913 | Intron 21 | T/A |  | 128/592 | 297/1241 | *0.385 (1.00)* | 0.90 (0.72-1.14) |  | 19/90/251 | 27/243/499 | ***0.042 (0.982)*** |
| PLXNA2 | rs6656034 | Intron 12 | A/G |  | 251/475 | 519/1023 | *0.668 (1.00)* | 1.04 (0.86-1.25) |  | 57/137/169 | 90/339/342 | *0.059 (0.996)* |
| PLXNA2 | rs7540179 | Intron 11 | A/G |  | 319/401 | 729/841 | *0.343 (1.00)* | 0.92 (0.77-1.10) |  | 83/153/124 | 166/397/222 | ***0.032 (0.958)*** |
|  |  |  |  |  |  |  |  |  |  |  |  |  |
| SEMA6A | rs41099 | Intron 4 | A/G |  | 129/585 | 221/1289 | ***0.038 (0.827)*** | 1.29 (1.01-1.63) |  | 18/93/246 | 20/181/554 | *0.076 (0.974)* |
| SEMA6A | rs258015 | Intron 3 | G/C |  | 212/490 | 390/1132 | ***0.024 (0.678)*** | 1.26 (1.03-1.53) |  | 37/138/176 | 54/282/425 | *0.072 (0.913)* |
| SEMA6A | rs258016 | Intron 3 | T/C |  | 174/542 | 313/1231 | ***0.030 (0.752)*** | 1.26 (1.02-1.56) |  | 25/124/209 | 36/241/495 | *0.100 (0.950)* |
|  |  |  |  |  |  |  |  |  |  |  |  |  |
| SEMA6B | rs10409783 | Intron 5 | A/G |  | 203/511 | 388/1198 | ***0.044 (0.867)*** | 1.23 (1.01-1.50) |  | 26/151/180 | 50/288/455 | *0.090 (0.941)* |
| SEMA6B | rs10422881 | Intron 3 | A/C |  | 196/518 | 372/1208 | ***0.045 (0.872)*** | 1.23 (1.01-1.50) |  | 24/148/185 | 44/284/462 | *0.107 (0.967)* |
|  |  |  |  |  |  |  |  |  |  |  |  |  |
| ^a^ Minor allele is shown first. All alleles are called on the forward strand. | | | | | | | | | | | | |
| ^b^ Based on 10,000 simulations | | | | | | | | | | | | |

**Supplementary Table 2: Significant results from allelic and genotypic association tests at *PLXNA2*, *SEMA6A* and *SEMA6B***

*Epistasis analysis:* Epistasis analysis was restricted to the seven SNPs that showed either allelic or genotypic association. SNPs within the same gene were not tested against each other. This analysis detected genetic interaction between rs2281913 (*PLXNA2*) and rs10409783 (*SEMA6B*) (Z = 8.634, *p = 0.003,* Supplementary Table 3). This result remains significant after Bonferroni correction for the 16 tests performed (*p = 0.05*). The increased risk detected by the interaction analysis is associated with carrying the AA genotype at rs2281913 and at least one copy of the A allele at rs10409783 (OR = 1.58, *p = 0.001, corrected p = 0.006*). Importantly, the epistatic interaction is more significant than the main effects at each SNP based on GAIA analysis (*p = 0.0027*).

|  |  | **rs10409783** | |
| --- | --- | --- | --- |
|  |  | **AA or AG** | **GG** |
| **rs2281913** | **AA** | 124 / 196  **OR = 1.58^a^** | 117 / 294  OR = 0.79^b^ |
|  | **AT** | 40 / 112  OR = 0.74^b^ | 47 / 123  OR = 0.80^b^ |
|  | **TT** | 6 / 15  OR = 0.86^b^ | 13 / 12  OR = 2.40^b^ |
| ^a^ 95% CI = 1.19, 2.09; *P = 0.001, corrected P = 0.006* | | | |
| ^b^ *P > 0.05* | | | |

**Supplementary Table 3: Case / control diplotypes and odds ratios for** **PLXNA2 (rs2281913) x SEMA6B (rs10409783)**

**Supplementary Methods**

**Human Sample Collection:** The SZ association sample consisted of 375 cases and 812 controls from the Republic of Ireland. Ethical approval for cases was obtained from the Federated Dublin Hospitals Ethics Committee, Adelaide & Meath Hospital, Incorporating the National Children's Hospital and for controls from the Trinity College School of Medicine Ethics Committee. Case individuals provided written informed consent and were interviewed by a psychiatrist or psychiatric nurse trained to use the Structured Clinical Interview for DSM (SCID-P) and Positive and Negative Symptom Scale (PANSS) rated on the basis of worst documented episode of illness. Diagnosis was made by the consensus lifetime best estimate method with DSM-IV criteria using all available information (interview, family or staff report and chart review). Cases met criteria for DSM-IV SZ (n=299) or schizoaffective disorder (n=76). All cases were over 18 years of age, of Irish origin (self reported Irish grandparents) and had been screened to exclude substance-induced psychotic disorder or psychosis due to a general medical condition. The case population was 64% male. The control sample, drawn from anonymized Irish blood donors, was 64% male and not specifically screened for psychiatric illness; donors were not taking regular prescribed medication as such individuals are excluded from blood donation in Ireland.

## SNP Selection – Bioinformatic Methods: All common variation (minor allele frequency (MAF) > 0.1) at SEMA6B (15.9kb in size; see below) was captured using a tag SNP selection method based on linkage disequilibrium (LD) measurements in the HapMap CEU sample, using Tagger as implemented in Haploview with an *r*^2^ threshold of 0.8 . The LD structure of the CEU sample is very similar to that of the Irish population making it a suitable reference panel . Due to the very large size of these genes (131kb, 217kb and 525kb respectively), SNP selection in *SEMA6A*, *PLXNA2* and *PLXNA4* was limited to variants located in the putative functional regions of each gene. Gene function maps were compiled using information mined from databases such as UCSC Genome Bioinformatics (http://genome.ucsc.edu/), ECR Browser (http://ecrbrowser.dcode.org/ ), Cluster Buster (http://zlab.bu.edu/cluster-buster/ ) and Genomatix (http://www.genomatix.de/). Each gene map contains positional information on all known exon structure and splice sites, evolutionarily conserved regions (ECRs), transcription factor binding sites (TFBS), CpG islands, other promoter elements and enhancer and silencer elements. SNP positions were overlaid on these maps, identifying SNPs located in putative functional regions of each gene.

*PLXNA2*: This gene spans 217kb on chromosome 1q32.2 and contains 32 exons. *PLXNA2* has previously been reported as associated with SZ and our analysis concentrated on the reported susceptibility region that extends from exon 5 to the 3’ end of the gene . Within this 70 kb region we genotyped the 4 associated SNPs from the study of Mah et al (X) (rs752016, rs841865, rs1327175 and rs2498028) and selected a further 11 SNPs that altogether captured ~65% of common variants in this region based on HapMap CEU data. We had 50-68% power to replicate the original associations based on reported odds ratios (ORs). However, our power to replicate is less than 8% when calculated based on the lower 95% confidence interval (CI) of these ORs. In addition, we genotyped 3 additional SNPs that represent the only known common missense polymorphisms in the gene (rs3748735, rs11119014 and rs2782948; all located in exon 2).

*PLXNA4*: This gene has 3 RefSeq isoforms (NM_020911, NM_181775, NM_001105543) that span a region of 525kb on chromosome 7q32.3. Nineteen SNPs were selected for analysis based on their positioning within putative functional regions of the gene.

*SEMA6A*: This gene extends over a distance of 131.2 kb on chromosome 5q21.3 and contains 19 exons. Intron 1 accounts for a large proportion of the gene (69.2 kb). A total of 23 SNPs were genotyped across the gene. Eighteen of these SNPs are located in the exon 2 – exon 19 region of the gene and effectively tag ~60% of all common HapMap SNPs in this region. The remaining 5 SNPs are located in ECRs of intron 1 and the putative upstream regulatory regions.

*SEMA6B*: This gene spans 15.9 kb on chromosome 19p13.3 and contains 16 exons. Ten tag SNPs captured all 14 common variants across the locus based on HapMap CEU data.

**Genotyping and Statistical Analysis:** SNPs were genotyped either by the Sequenom iPlex™ system (Dept. of Psychological Medicine, Cardiff University), by Kbiosciences (Hoddesdon, UK) using a KASPar assay or by Custom Taqman assay on an ABI PRISM 7900HT Sequence Detection System in our laboratory. Details of all genotyping assays are available on request. A total of 74 SNPs were genotyped in the case-control sample. Two SNPs, rs6662281 in *PLXNA2* and rs7807933 in *PLXNA4*, were excluded from analysis due to low call rate (69.2%) and lack of Hardy-Weinberg Equilibrium (HWE; p < 0.001) respectively. The remaining 72 SNPs were in HWE (p > 0.001) and had an average call rate of 95.7%. Comparison of genotypes between our data and HapMap indicated a genotyping accuracy of >99%. DNA samples from the HapMap CEU sample were randomly distributed in our case-control DNA plates. This permitted the cross-referencing of online data with genotype data generated during this study for the purposes of quality control. SNPs were tested for association with the phenotype by allelic (2x2) and genotypic (3x2) tests using PLINK . Haplotype association analysis was performed across each locus with 2- and 3-marker sliding windows using PLINK to produce omnibus tests for each marker combination and tests of each individual haplotype. Epistasis analysis was performed using PLINK.

*Epistasis analysis:* Epistasis analysis was restricted to the seven SNPs that showed either allelic or genotypic association. SNPs within the same gene were not tested against each other. This analysis detected genetic interaction between rs2281913 (*PLXNA2*) and rs10409783 (*SEMA6B*) (Z = 8.634, *P = 0.003)*. This result remains significant after Bonferroni correction for the 16 tests performed (*P = 0.05*). No case sample was identified to have the rare homozygous genotype at both loci. Therefore, the rare homozygous genotype for rs10409783 (AA) was coupled with the heterozygous genotype (AG) for the calculation of ORs for each diplotype. The increased risk detected by the interaction analysis is associated with carrying at least one copy of the A allele at rs10409783 and carrying the AA genotype at rs2281913 (OR = 1.58, *P = 0.001, corrected P = 0.006*). These represent the risk allele and the risk genotype at rs10409783 and rs2281913, respectively, based on the single SNP analysis. Importantly, the epistatic interaction is more significant than the main effects at each SNP based on GAIA analysis (*P = 0.0027*).

**Supplementary References**

**1. Mah S, Nelson MR, Delisi LE, Reneland RH, Markward N, et al. (2006) Identification of the semaphorin receptor PLXNA2 as a candidate for susceptibility to schizophrenia. Mol Psychiatry.**

**2. Barrett JC, Fry B, Maller J, Daly MJ (2005) Haploview: analysis and visualization of LD and haplotype maps. Bioinformatics 21: 263-265.**

**3. de Bakker PI, Burtt NP, Graham RR, Guiducci C, Yelensky R, et al. (2006) Transferability of tag SNPs in genetic association studies in multiple populations. Nat Genet 38: 1298-1303.**

**4. O'Dushlaine CT, Dolan C, Weale ME, Stanton A, Croke DT, et al. (2008) An assessment of the Irish population for large-scale genetic mapping studies involving epilepsy and other complex diseases. Eur J Hum Genet 16: 176-183.**

**5. Ovcharenko I, Nobrega MA, Loots GG, Stubbs L (2004) ECR Browser: a tool for visualizing and accessing data from comparisons of multiple vertebrate genomes. Nucleic Acids Res 32: W280-286.**

**6. Frith MC, Li MC, Weng Z (2003) Cluster-Buster: Finding dense clusters of motifs in DNA sequences. Nucleic Acids Res 31: 3666-3668.**

**7. Purcell S, Neale B, Todd-Brown K, Thomas L, Ferreira MA, et al. (2007) PLINK: a tool set for whole-genome association and population-based linkage analyses. Am J Hum Genet 81: 559-575.**
